# The Effect of Antimony (III) Oxide on the Necessary Amount of Precursors Used in Laser-Activated Coatings Intended for Electroless Metallization

**DOI:** 10.3390/ma15155155

**Published:** 2022-07-25

**Authors:** Bartłomiej Jagodziński, Piotr Rytlewski, Krzysztof Moraczewski, Andrzej Trafarski, Tomasz Karasiewicz

**Affiliations:** Institute of Materials Engineering, Kazimierz Wielki University, 85-064 Bydgoszcz, Poland; prytlewski@ukw.edu.pl (P.R.); kmm@ukw.edu.pl (K.M.); trafarski@ukw.edu.pl (A.T.); tomakara@ukw.edu.pl (T.K.)

**Keywords:** surface activation, electroless metallization, organometallic complex, polymers

## Abstract

The article presents research on the potential use of organometallic compounds with the addition of antimony (III) oxide Sb_2_O_3_ as a coating additive that will make coatings susceptible to electroless metallization after prior surface irradiation with 193 nm wavelength laser radiation and a different number of laser pulses. The surface modification and activation effects were assessed by optical-imagining as well as by scanning electron microscopy (SEM) with energy dispersive analysis (EDX). It was found that the presence of Sb_2_O_3_ in the coating made it possible to reduce the content of the copper complex, causing an intensive surface ablation, resulting in the formation of a conical structure with a higher content of metallic copper nuclei.

## 1. Introduction

A very important aspect for the application of polymeric materials in the electronics industry is their electrical conductivity. Polymeric materials have very little or no electrical conductivity [1,2]. For this group of materials to be electrically conductive, their surface should be covered with electrically conductive metal [3]. The deposition of metal on the surface of the polymeric material usually is conducted in an electroless process. In the classic electrolessmethod, the entire surface of the polymer is activated by immersing the sample in the activation solution. These solutions contain various types of chemical compounds, such as strong oxidants, acids and solvents. The most commonly used are solutions of sodium dichromate (VI), potassium dichromate (VI) and potassium madmanganate (VII) in dilute sulfuric acid (VI) [4,5]. Phenol, sodium hydroxide, sodium hypochlorite and metallic sodium are also used [6], which negatively affect the environment. Electroless metallization is usually a multi-step process in which several different baths for various purposes are used.

Selective surface activation and metallization are required for polymeric materials to be used in the electronics industry. However, in chemical activation methods, which are most often used in the case of polymeric materials, the entire surface is activated, therefore, the selectivity of metallization is achieved by covering the surface with the so-called masks. This procedure causes additional technological difficulties, increasing the time and cost of the entire metallization process. For these reasons, new techniques for selective surface activation of polymeric materials are being developed, e.g., through the use of lasers and their ability to modify only irradiated areas [7,8]. The use of lasers makes it possible to eliminate the use of several baths for surface activation, and thus the use of concentrated and hazardous chemicals. However, to successfully activate the surface of polymeric materials by laser irradiation, a suitable catalyst should be incorporated into the matrix of the polymeric material or a coating with this catalyst (precursor) should be applied [9,10]. A whole group of compounds is known that can act as a precursor for electroless metallization. The precursors (catalysts) can be inorganic or organic metal compounds. Most often palladium, copper, zinc, silver or gold compounds in the form of acetates, acetylacetonates or complexes are used [8,11,12,13,14,15].

The laser radiation acting on the surface of the polymeric material causes the organic ligands of organometallic compounds to ablate together with the polymer matrix. The heavier metal atoms remain on the surface and are simultaneously reduced to a metallic species. These species are the active (catalytic) sites for the further reduction of metal ions present in the bath intended for metallization [16].

The most common disadvantage of the described metallization process using organometallic precursors is the relatively large amount of the precursor used in the coating or matrix of polymeric material. To solve this problem, compounds that absorb ultraviolet (UV) or infrared (IR) laser radiation can be additionally incorporated into the coatings. The addition of this types of compounds to the coatings makes it possible to reduce the necessary content of the electroless metallization precursor, while maintaining very similar metallization results [12].

The efficiency of precipitation of the active metal centers of electroless metallization from the precursor compounds under the influence of laser radiation can be additionally increased by the use of radiation absorbers, which improve the conditions for the precipitation of metallic species. As shown in previous studies, the addition of glass fibers or microspheres positively influenced the effectiveness of surface activation [17,18].

This paper presents the effect of antimony (III) oxide as an additional component of coatings on the results of laser activation of coatings intended for electroless metallization. It has been shown that the addition of antimony (III) oxide to polyurethane coatings has a positive effect on the effects of electroless metallization. The use of this additive made it possible to reduce the content of copper (II) acetylacetonate Cu(acac)_2_ in the coating by a half, while obtaining comparable metallization effects.

## 2. Materials and Methods

### 2.1. Materials

The following materials were used in the research:

- Polinitrate (V) complex of [(2-amino-5-guanidine-pentane) (mi-O,O′-nitrate(V)) (2,2′-dipyridile) copper (II)]{[Cu(μ-*O*,*O*′-NO_3_)(l-arg)(2,2′-bpy)]·NO_3_}n abbreviated as compound A;
- Copper (II) acetylacetonate Cu(acac)_2_ (Sigma Aldrich, Saint Louis, MO, USA) abbreviated as compound B;
- Antimony (III) oxide Sb_2_O_3_ (particle size < 250 nm) (Sigma Aldrich, Saint Louis, MO, USA);
- Polyurethane resin B4060 (Haering, Bubsheim, Germany);
- Polycarbonate (PC) Xantar 19 UR (DSM Engineering Plastics, Holand);
- Autocatalytic metallization bath M-Copper-85 (MacDermid, Łysomice, Poland);
- Formaldehyde HCHO, 36% (POCH, Gliwice, Poland), molecular weight 30.03 g mol^−1^;
- Adhesive Araldite 2011 (Huntsman, Basel, Switzerland).

Polyurethane coatings containing two types of precursors (A and B) and an Sb_2_O_3_ absorber were applied to polycarbonate plates, which had been produced by an industrial injection-molding process.

Complex A is not commercially available and has been synthesized in a crystalline form. More information about the compound is provided in the patent [19]. In the presented research, the main objective was to verify whether this compound can be used as an effective laser-activated precursor, at the same time comparing the results with the precursor B [12].

The total content of the additives used in the polyurethane coating was 20 wt.%, and the coatings contained A or B complex and Sb_2_O_3_. The composition of tested coatings and their designations are listed in Table 1.

The applied coating ingredients were mechanically mixed with a polymer resin, evenly applied by pouring onto a polymer substrate made of PC and leveled with a flat applicator. The thickness of the applied coatings ranged from 300 to 700 µm.

### 2.2. Laser Irradiation and Metallization

The coatings were irradiated with a pulsed ArF excimer laser that emits ultraviolet radiation (λ = 193 nm). The coatings were irradiated with 300, 400 or 500 laser pulses with a fluence of 100 mJ/cm^2^. Excimer laser ArF (λ = 193 nm) was selected as its photon energies (6.4 eV) are high enough to break any chemical bonds in organic compounds.

After surface activation, electroless metallization was carried out using a six-component M-Copper 85 bath (MacDermid-Poland, Łysomice, Poland) with formaldehyde as a reducing agent. The metallization was carried out at the temperature of 46 °C, with the pH of the solution 12.8.

### 2.3. Methodology

The surface topography of the tested coatings changes under the influence of laser radiation and electroless metallization effects were examined with the Phenom XL scanning electron microscope (SEM) (Thermo Fischer Scientific, Eindhoven, The Netherlands). Before testing, the samples were sprayed with a gold layer of about 2 nm thickness.

The contact-angle measurements were performed using a DSA 100 goniometer (Kruss, Germany). The wettability tests were carried out about 24 h after the laser irradiation. During the tests, droplets with the measuring liquid (water or diiodomethane) were deposited on the surface of the coatings. The volume of droplets was increased at the rate of 5 μL/min, by automatically measuring the contact angle every second. Based on the determined mean values from approximately 100 measurements of the contact angle for each water and diiodomethane droplet, the surface energy (SE) of the tested coatings was calculated using the Owens–Wendt method [20]. In this method, the SEP is divided into polar ($\gamma_{S}^{p}$) and dispersion ($\gamma_{S}^{d}$) components and the value of SE was determined from the following equations:

$$\left\{\begin{array}{l} {\left(\gamma_{S}^{d}\right)}^{1/2}+1.53{\left(\gamma_{S}^{p}\right)}^{1/2}=7.80(1+\cos\theta_{w})\\ {\left(\gamma_{S}^{d}\right)}^{1/2}+0.22{\left(\gamma_{S}^{p}\right)}^{1/2}=3.65(1+\cos\theta_{d}) \end{array}\right.$$

where: θ_w_—water contact angle, θ_d_—diiodomethane contact angle.

Tests of the adhesive strength of coatings and the substrate as well as the deposited copper layer and the coating were carried out by tearing off the glued stamp with the Instron 3367 testing machine (Instron, Norfolk County, MA, USA). The metal stamp was glued to the copper layer with Araldite 2011 (Huntsman, Basel, Switzerland) adhesive. After approximately 48 h, the sample with a metal stamp was clamped in specially designed clamps. Measurements were carried out at a tensile speed of 2 mm/min. The adhesive strength was calculated as the maximum force exerted on the surface of the stamp glued to the coating. The area of the stamp glued to the metallic layer was 130 mm^2^ (6.5 × 20 mm).

## 3. Results

**Figure 1:**
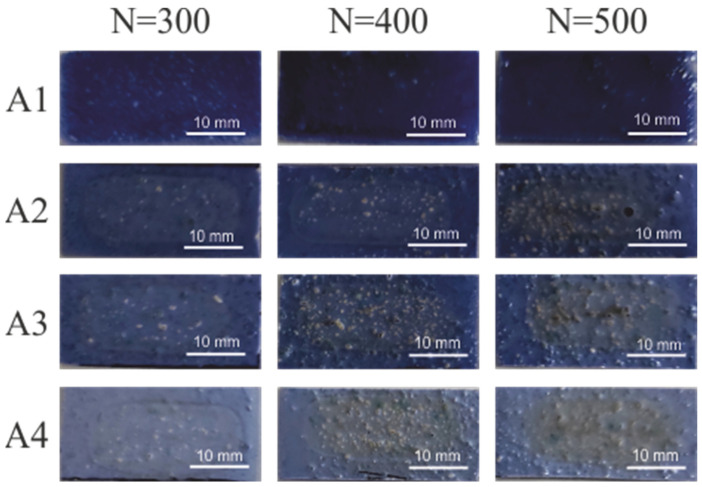
The effects of electroless metallization of A1–A4 coatings.

The coatings with the composition given in Table 1 were subjected to laser irradiation with a different number of pulses and fluence in accordance with the parameters given in the methodology, and then electroless metallized. The effects of electroless metallization of coatings containing compound A with Sb_2_O_3_ are presented in Figure 1.

As shown in Figure 1, none of the coatings was electroless metallized with copper. This may prove that the amount of copper precipitated from the A precursor is insufficient for effective electroless reduction of copper ions contained in the metallization bath. The lack of copper plating may be due to the low mass fraction of copper in precursor A (12.8%), which is significantly less than for precursor B (about 24%). This result can be a valuable guide in the search and selection of new compounds as precursors in this types of metallization techniques. Nevertheless, this factor is certainly non-exclusive, and the efficiency of precipitation and agglomeration of local copper agglomerates may be influenced by the structure of organic ligands and their susceptibility to laser radiation.

**Figure 2:**
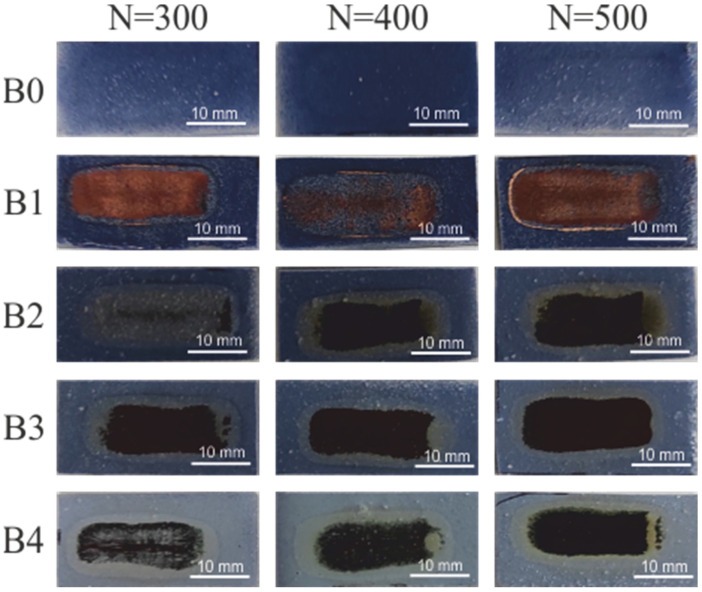
The effects of electroless metallization of B0–B4 coatings.

Figure 2 shows the effects of electroless metallization of irradiated coatings containing compound B and Sb_2_O_3_.

Coating B0, containing 10 wt.% of complex B, after laser irradiation and electroless metallization, was not covered with a copper layer. When increasing the content of this complex to 20 wt.%. (coating B1) a copper coating appears on the surface of the coating. Additionally, when using a smaller amount of complex B but with the addition of Sb_2_O_3_, it can be seen that the samples are coated with a dark copper layer. Thus, a clear influence of the presence of Sb_2_O_3_ on the effects of electroless metallization is visible. The same amount of compound B (10 wt.%) is present both in the B0 and B4 coatings, but a copper layer was obtained only in the case of B2 coating. The presence of the absorber has a positive effect on the effects of laser irradiation and electroless metallization. Practically the entire laser-irradiated surface of the coatings was covered with a layer of copper after the electroless metallization process. The dark color of copper may result from its rapid oxidation in the metallization solution while it is still being immersed. Interestingly, the dark copper layers were characterized by a very good electrical conductivity (0.24 S/cm) compared to that of the B1 coating (8.7 × 10^−9^ S/cm).

**Figure 3:**
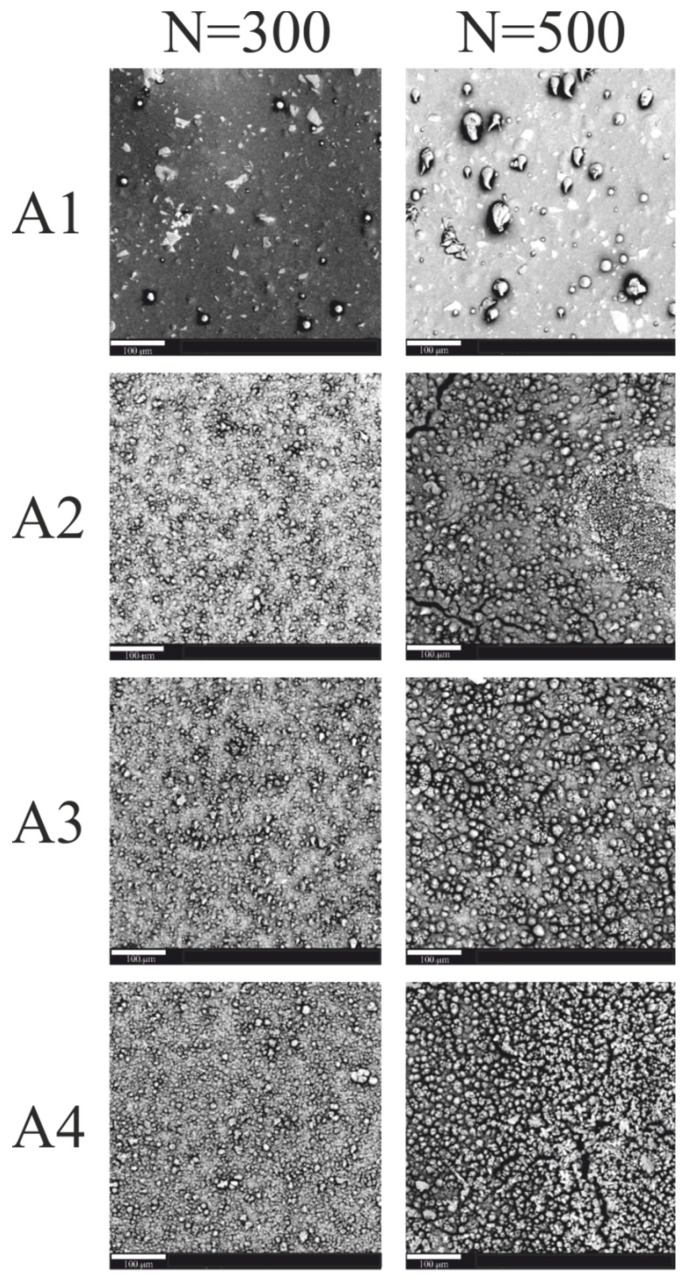
SEM images of A1–A4 coatings (scale—100 μm).

The effects of irradiation of coatings with a pulsed excimer laser ArF are shown in the SEM images (Figure 3).

The SEM images in Figure 3 show the surface of the coatings irradiated with 300 and 500 laser pulses of the same unit energy (100 mJ/cm^2^). There is a visible change in the surface topography of the irradiated coatings. The effect of laser irradiation on the coatings is the appearance of more local aggregates on the surface of samples containing nanoparticles of the Sb_2_O_3_ absorber. The exposed Sb_2_O_3_ particles visible in the SEM are due to laser ablation of the coating matrix. Probably, no copper was precipitated on the surface of the irradiated coatings, which resulted in the lack of deposited copper layer in the electroless metallization process. The number of visible aggregates increases with the increase in the number of laser pulses.

**Figure 4:**
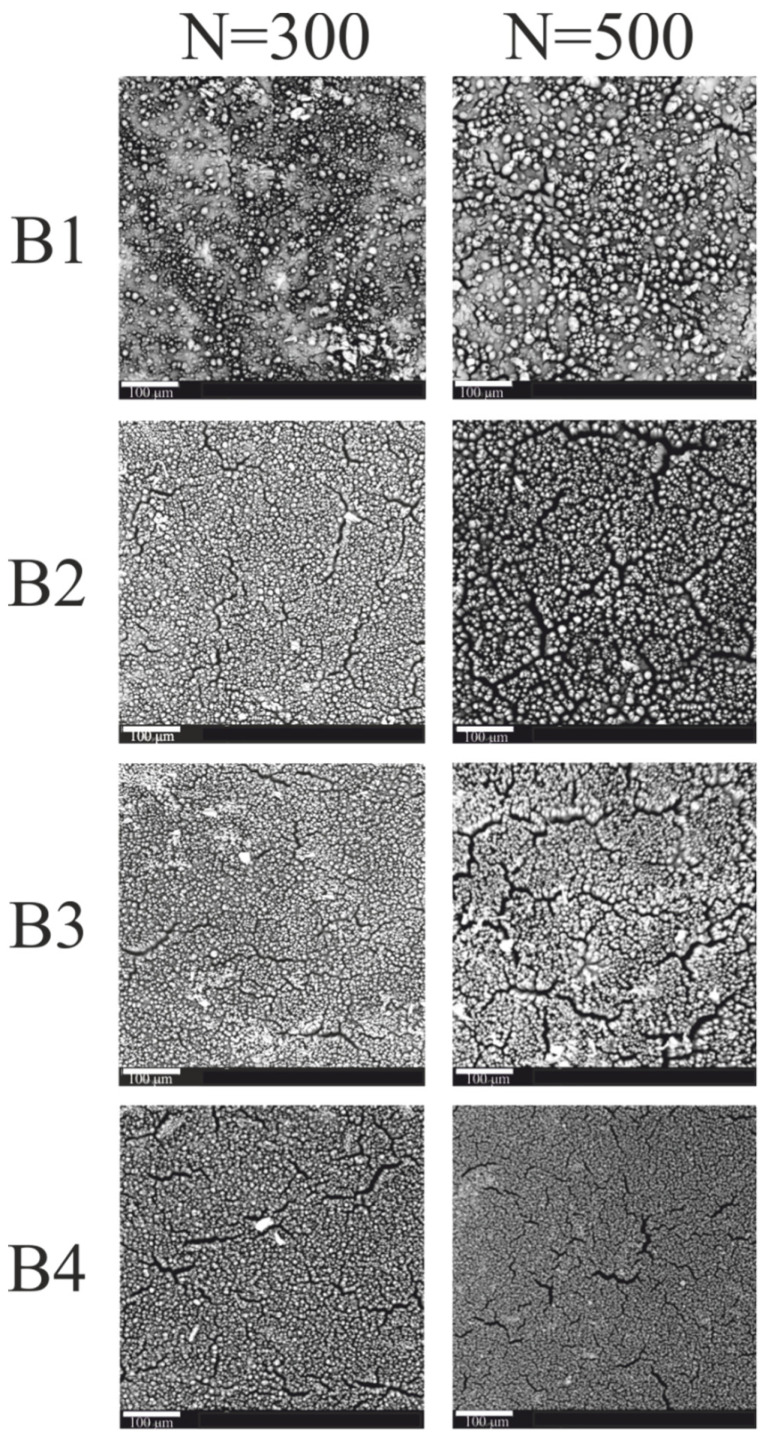
SEM images of B1–B4 coatings (scale—100μm).

Bright areas are visible in the recorded images of the B coatings (Figure 4). The number and size of these areas increased with the increase in the number of laser pulses. The bright areas visible in the SEM images correspond to the places where the electrons are intensely emitted, the source of which can be precipitated copper.

Additionally, in the case of coatings with compound B, the greater the number of laser pulses, the more differentiated the surface topography. Furthermore, numerous cracks are visible on the surface of the irradiated coatings at 300 and 500 laser pulses.

**Figure 5:**
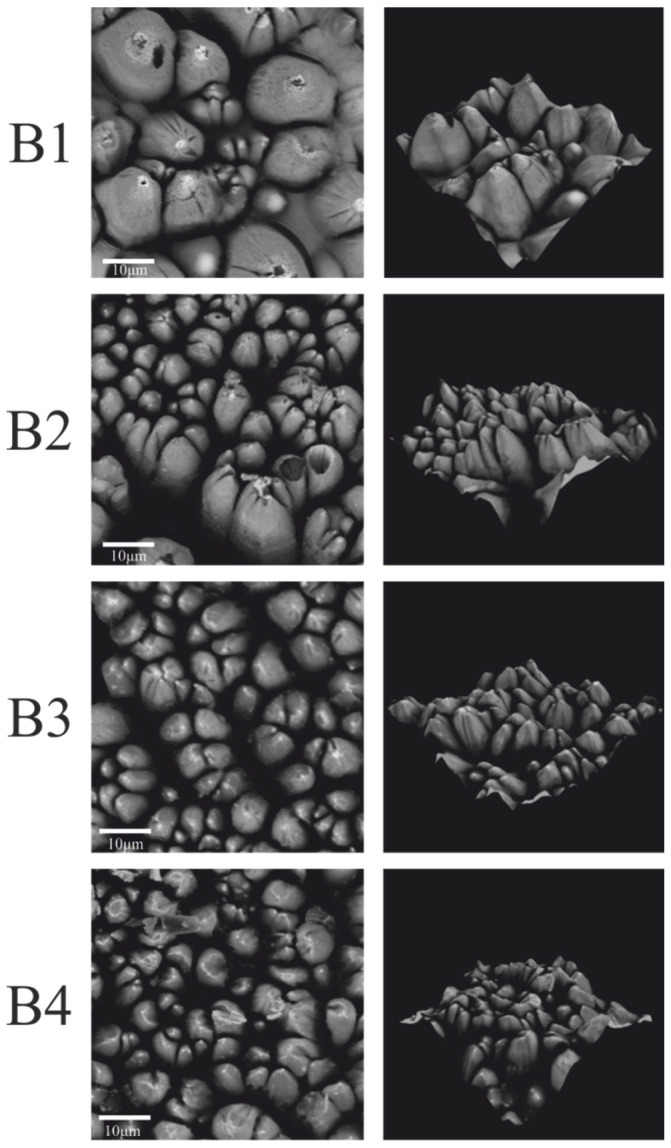
SEM images of B1–B4 coatings (N = 500, scale—10 μm).

At a higher magnification of the surface of samples B (Figure 5), a very interesting conical surface structure was revealed. The lower number of cones was formed on the surface of B1 coating as a result of laser radiation. On the B2, B3, and B4 coatings, a much larger number of cones were visible. The formed cones varied in shape and size. It has been noticed that the greater the amount of absorber (Sb_2_O_3_) in the coating, the denser the conical structure, i.e., the resulting cones are closer to each other. Numerous cracks can be seen between the formed cones. This may be due to the degassing of the coating components. Air bubbles may have formed during the application of the coatings or during the cross linking of the resin. Then, during laser irradiation and the ongoing ablation, air bubbles could be released, which resulted in cracks on the surface of the coatings and defects in the structure of the cones.

By analyzing Figure 4 and Figure 5, it can be seen that the number of laser pulses also had an impact on the formation and structure of the conical structures. With the same coating composition, more cones are formed as the number of laser pulses increased. The resulting cones are of various heights and shapes. One can observe cones that are very tilted, cones with open tops, and cones with cut tops. This is most likely the result of the described intense degassing process, which also takes place in that cone structures. It should be mentioned that a similar phenomenon occurs when the absorber content in the coating increased.

Based on the SEM and EDX studies, the causes of the formation of a conical surface can be formulated. The ablation process of the surface layer of the coatings occured under the influence of laser radiation. For most polymeric materials, the ablation threshold for 193 nm laser radiation is less than 20 mJ/cm^2^ [21]. However, the copper ablation threshold for laser radiation of the same wavelength is much higher, over 2 J/cm^2^ [22]. The ablation threshold of antimony also is higher than that of polymeric materials because that element was detected on the surface of the coatings. The entire surface of the cones after laser irradiation is covered with antimony, where the greatest amount was detected at the tops of the cones. The presence of antimony is confirmed by the results presented in Table 2.

In the research, laser pulses with a unit energy of 100 mJ/cm^2^ were used. Such radiation will ablate the polymer matrix, but the locally agglomerated copper will remain on the coating surface. The local copper agglomerates will thus act as a protective mask for the polymeric material situated below these agglomerates. With successive laser pulses, the material is etched from the areas between the agglomerates, creating a characteristic structure, and the height of the cones increases.

Due to the locally different structure of the surface layer of the irradiated coatings, the intensity of X-ray radiation corresponding to copper was investigated using the EDX method from the largest possible scanning area. The results of these tests are presented in Table 2.

The amount of precipitated copper on the surface of the coatings increases with the increase in the content of compound B in the coatings, both for the number of pulses of 300 and 500. The copper content for compound B ranged from 4% for coating B4 irradiated with 300 pulses to almost 10% for coating B1 irradiated with 500 pulses. The different content of the copper precipitated on the coating surface may be because copper agglomerates occurred mainly locally on the surface of the tops of formed cones. However, even the smallest amount of precipitated copper made it possible to effectively metallized samples and obtain a metal layer.

In the case of coatings containing antimony, copper was only detected after more intense laser irradiation (N > 300). The highest amounts of antimony were detected on the surface of the formed cones, especially at the tops, where the content of this element reached 30 at.%.

**Figure 6:**
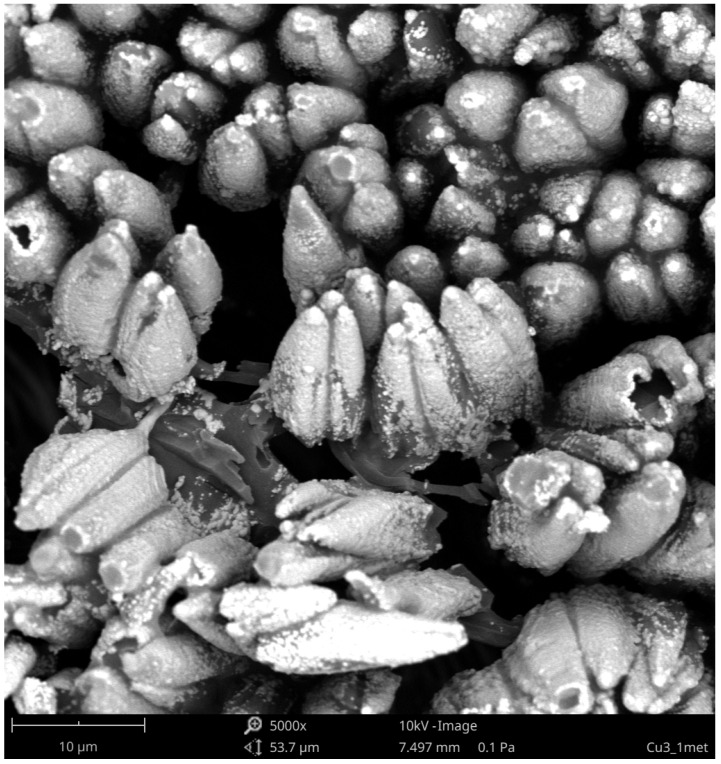
SEM image of metallized B3 coating (N = 300).

The microscopic effects of electroless deposition of the selected coating are shown in Figure 6.

After the electroless metallization process, the structure of the deposited copper reflected the conical structure formed during laser irradiation. Practically the entire surface of the B3 coating was covered with a copper layer. The surface between the cones was covered with copper to a lesser extent, because there was much less copper precipitated during laser irradiation. The shape of the cones tops differed after irradiation and after the electroless metallization process. The tops of the cones became rounded after the metallization, which may indicate that the copper ions from the metallizing bath were reduced more intensively at the tops of the cones than between them. A greater amount of deposited copper on the cones tops may indicate a much greater amount of copper precipitated during laser irradiation, as well as the formation of their local agglomerates. The thickness of the obtained copper layer after electroless metallization was in the range from 8 to 25 μm, but the thicker part was located around the center of laser spot.

After metallization, the same defects in the cone tops as after laser irradiation are noticeable. This may indicate that no copper was precipitated at the tops of these cones. There are also light areas on the tops of the damaged cones, indicating more copper precipitation during laser irradiation, which led to an increase in the amount of electroless deposited copper.

**Figure 7:**
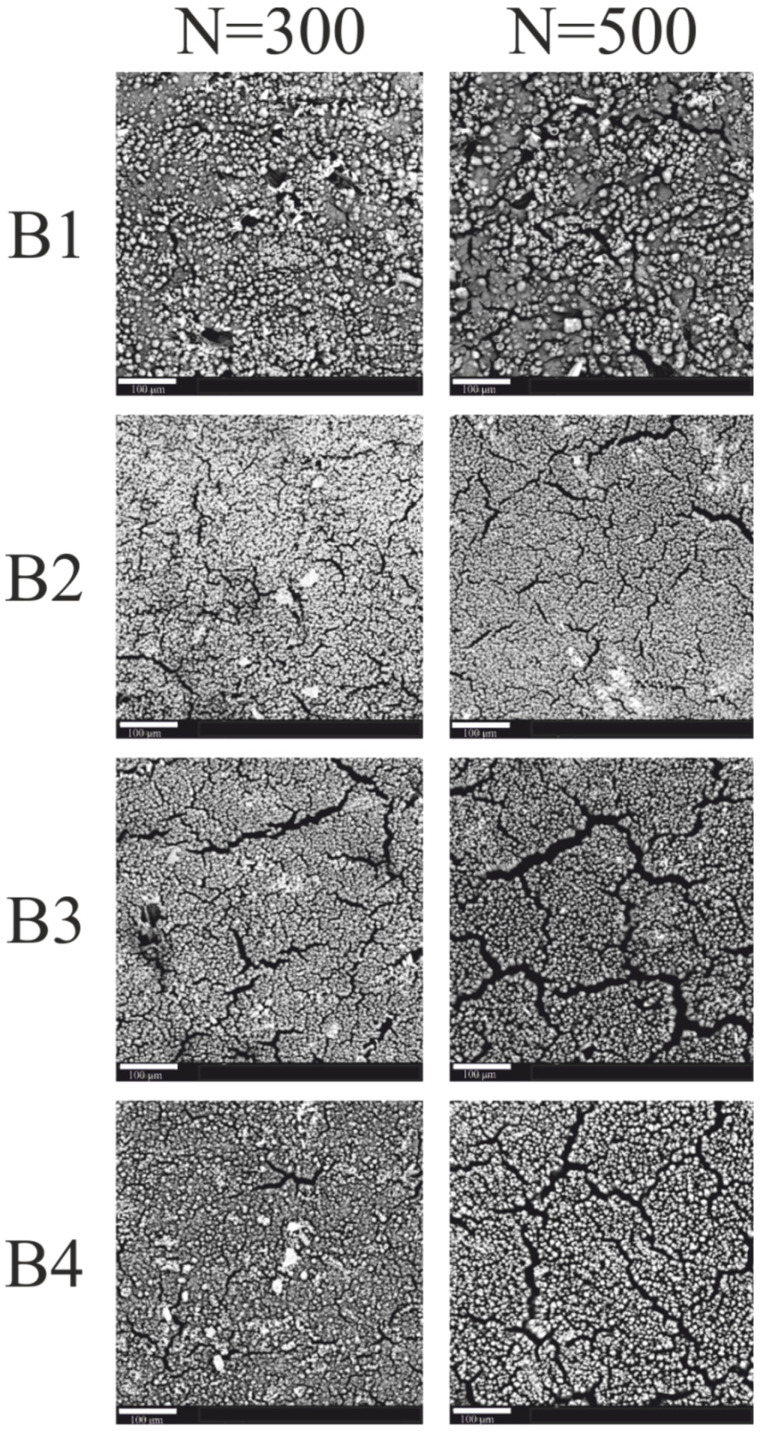
SEM images of metallized B1–B4 coatings (scale—100 μm).

The surface structure of the metallized coatings reflects the surface structure after irradiation, as shown in Figure 7. As a result of electroless metallization, the entire surface was covered with a layer of copper. However, numerous cracks are visible on the surface of the coatings as after the irradiation process. However, the copper layer is not uniform, as numerous cracks are visible on the surface of the coatings as after the irradiation process.

Table 3 shows the results of the surface-energy calculations. The surface-energy calculations were performed using the Owens–Wendt method on the basis of measurements of water and diiodomethane contact angles. The results of surface energy calculations allow to assess the surface thermodynamic state of coatings after the irradiation process. Most of the non-irradiated coatings have a SE of about 40 mJ/m^2^. The laser modification increased the SEP value. In contrast, the number of laser pulses did not cause a significant increase in the SE value. The main factors influencing the changes in SE are the chemical composition and the geometric structure of the surface [23,24]. The increase in surface energy can lead to better adhesive properties of laser-modified coatings. However, caution should be taken when predicting adhesive properties only from wettability measurements and SE calculations.

The adhesive strength of the electroless-deposited copper layer was tested by the pull-off method with the use of a metal stamp and a tensile testing machine. In all cases, the copper layer could not be peeled off from the polymer coating. Based on the research on the adhesion of the copper layer to the surface of the coatings, it can be concluded that the adhesive strength is in the range of 2.4–3.1 MPa. It is believed that the high adhesive strength of the copper layer is due to the conical surface structure of the coating created under the influence of laser irradiation, which allows very good anchoring of the copper layer and the large contact surface with the adhesive through which the stamp was attached.

## 4. Conclusions

This paper compares two complex compounds considered as potential precursors of electroless metallization. The positive effect of the presence of antimony (III) oxide Sb_2_O_3_ on the effects of laser irradiation as well as electroless metallization was demonstrated. This compound made it possible to reduce the content of the organometallic metallization precursor. The conducted experiments proved that complex B was an effective precursor of electroless metallization. The additional presence of the Sb_2_O_3_ in the coating caused greater changes in the surface topography under the influence of ultraviolet laser radiation, absorbing the radiation incident on the surface of the coatings. SEM images showed that a rough surface structure with visible cones was formed in the irradiated areas. A much greater number of precipitated cones occurred on the coatings with compound B. Additionally, copper precipitated on the surface of the irradiated coatings with compound B, which constituted active catalytic centers for electroless metallization. The precipitated copper was detected locally, especially on the surface of the cones. The deposited copper layer on the coatings with compound B was characterized by a very good adhesive strength exceeding 2 MPa and an electrical conductivity of about 0.24 S/cm.

## Figures and Tables

**Table 1:** Composition of coatings.

Coating	Content of Compounds: [wt.%]		
	Complex A	Complex B	Sb_2_O_3_
A1	20	-	-
A2	16.67	-	3.33
A3	15	-	5
A4	10	-	10
B0	-	10	-
B1	-	20	-
B2	-	16.67	3.33
B3	-	15	5
B4	-	10	10

**Table 2:** The content of Cu, O, C and Sb atoms in the surface layer of the tested coatings irradiated with a different number of laser pulses.

Nazwa Próbki	N = 300				N = 500			
	Cu at. [%]	O at. [%]	C at. [%]	Sb at. [%]	Cu at. [%]	O at. [%]	C at. [%]	Sb at. [%]
A1	-	30.45	69.55	-	0.41	29.84	69.45	-
A2	0.36	27.90	64.75	-	2.38	24.69	67.85	5.07
A3	0.46	29.13	70.41	-	1.46	21.33	68.05	9.16
A4	0.19	29.83	69.97	-	1.19	25.14	63.93	9.74
B1	7.37	21.18	71.45	-	9.40	21.48	69.12	-
B2	4.94	20.91	74.14	-	7.10	23.66	67.54	1.70
B3	7.92	20.83	67.62	3.62	7.64	22.28	64.05	6.02
B4	4.03	19.75	59.08	17.14	4.86	25.11	55.65	14.38

**Table 3:** Results of surface energy calculations.

Coating	Surface Energy [mJ/m^2^]		
	Unmodified	Modified N = 300	Modified N = 500
A1	42.7	45.3	42.9
A2	38.3	55.2	61.2
A3	47	50.8	59.1
A4	42.1	51.5	68
B1	42.1	82.6	74.8
B2	38.6	86.4	75
B3	37.3	67.5	75
B4	72.8	78.8	77.1

## Data Availability

Not applicable.
